# Increasing Engagement for Breast Cancer Screening and Treatment: The “ICANTREAT” Community of Expertise Initiative

**DOI:** 10.31557/APJCP.2020.21.12.3655

**Published:** 2020-12

**Authors:** Thejas R Kathrikolly, Suma Nair, Amudha S Poobalan, Ranjitha S Shetty, Sheela Tripathee, Sara J Mac Lennan

**Affiliations:** 1 *Department of Community Medicine, Kasturba Medical College, Manipal*,* Manipal Academy of Higher Education (MAHE), Manipal, Karnataka, India. *; 2 *Centre for Community Oncology, KMC, Manipal, India. *; 3 *Institute of Applied Health Sciences, University of Aberdeen, Scotland, *; 4 *Centre for Indigenous Population, KMC, Manipal, India. *

**Keywords:** Stakeholder, workshop, cancer, care, patient advocate

## Abstract

**Background:** Incidence of breast cancer and associated mortality are on the rise globally. Breast cancer incidence in India is on the rise and also accounts for a staggering 50% mortality rate among women. Health illiteracy, socio-economic and cultural barriers in addition to lack of an organized nationwide screening and prevention programme contribute to poor patient outcomes. Thus, it is imperative to strengthen the existing screening and treatment facilities to address the increasing cancer burden. In this regard, we conducted a workshop to investigate the scope of a multi- stakeholder engagement in breast cancer screening and treatment. **Methods:** A stakeholder workshop grounded in an established co-design methodology was convened in a semi-urban coastal district in South India with active participation of physicians, surgeons, occupational therapists, community leaders, programme officers, public health professionals and breast cancer survivors. Shiffman and Smith’s framework was adapted to highlight barriers to screening and role of stakeholders in the co-design of solutions. **Results:** Deliberate and active discussions marked the workshop proceedings resulting in the identification of individual and systems level barriers, facilitators and implementation strategies. Social stigma and non-existent standard protocols for screening and referral were recognised as critical barriers, while community engagement and a dedicated patient advocate were the proposed facilitators**.Conclusion: **This workshop was an important step in bringing together key stakeholders and marked the beginning of the ICANTREAT initiative and a Community of Expertise. The outcomes highlight the importance of stakeholder involvement in the cancer- care pathway for breast cancer screening, diagnosis and treatment.

## Introduction

Breast cancer is one of the most common cancers among women worldwide contributing to 11.6% of all new cases, 6.6% of all cancer deaths and 15% of all female cancer deaths (World Health Organization, 2012). According to GLOBOCAN 2018, between 2012 and 2018, there was a considerable increase in the incidence of breast cancer (from 1,700,000 to 2,089,000 cases) as well as mortality (from 522,000 to 627,000) (GLOBOCAN, 2018). Of the total cases of breast cancer, 53% are reported from low and middle-income countries (LMICs) (Fitzmaurice et al., 2015). Asian countries, which represent 59% of the global population, account for 39% of the new breast cancer cases and 44% of deaths (Fan et al., 2015). 

Increased incidence of breast cancer is breaking the urban rural divide in India as the disease has been on the rise with an age standardized incidence rate of 41.0 per 100,000 (Manoharan et al., 2017) and a case fatality rate of nearly 50% (Breast Cancer India, 2018). India’s coverage of population-based cancer registries is among the lowest in the world (FICCI Ladies Organization (FLO) with Ernst and Young LLP (EY), 2017) and cancer statistics based on these cancer registries could be a gross under estimation of the cancer burden. Studies report a higher incidence among younger women in the age group of 35-40 years, in whom cancer tends to be more aggressive in nature. India accounts for a high mortality-to-incidence ratio, where for every two women diagnosed with the disease, one dies of it. In some rural areas this ratio is observed to be as high as 66% (Malvia et al., 2017). Health illiteracy, socio-economic and cultural barriers coupled with a lack of organized nationwide screening and prevention programs has resulted in delayed presentations of the disease leading to poor patient outcomes (Khokhar, 2012).

A dearth of evidence-based guidelines and algorithms that aid early disease detection and a lack of prompt referral for additional care, both contribute to loss of time and resources (Nandakumar et al., 2010). In addition, recent studies have highlighted the lack of support, poor co-ordination and lack of information systems for breast cancer in LMICs (Rivera-Franco and Leon-Rodriguez, 2018). There has been an increasing demand for strengthening the existing screening and treatment facilities in India to address the rising cancer burden (Suhag et al., 2015). The strategies should include needs assessment, identification of people’s perception, and consequently integrating them with measures for early detection, clear pathways of care and affordable treatment facilities. These measures are vital for reducing incidence and mortality of breast cancer among Indian women. 

This paper presents the results of a multi-stakeholder workshop undertaken to investigate the scope of engagement in breast cancer screening, diagnosis and treatment in India. This holistic system understanding is currently lacking in behavioural interventions and health services design and delivery within this setting.

## Materials and Methods

*Study Design*

The design of the workshop was grounded in an established co-design methodology (Donetto et al., 2015). One of the key strengths of this co-design methodology is that the identified research and change areas are perceived to be more applicable and acceptable to the end-users of the research (Slattery et al., 2020). When conducted well, comprehensive and effective stakeholder involvement can be realised, which is an important aspect of a robust methodology. The detailed description of methods for designing and conducting this study speaks to its rigour and can in turn facilitate successful outcomes of future interventions.

*Reinforcing the stakeholder involvement in the care pathway (diagnosis and treatment) of breast cancer*

Stakeholder involvement in the design and delivery of the clinical care pathway, from screening through to diagnosis and treatment of breast cancer is imperative to ensure implementation of key behaviours, engagement and positive outcomes for the individuals with cancer (Barger et al., 2019). The development of a network (real or virtual) between various stakeholders can further strengthen the development and implementation of clinical care pathways as demonstrated in other conditions and contexts (MacLennan et al., 2011). This involvement needs to be framed by an appropriate process and the contributions of each of the stakeholder groups managed and understood at key stages across the cancer journey. With this in mind, an expert group workshop was convened in a semi-urban coastal district in South India with 15 key stakeholders, to map the facilitators and barriers to breast cancer screening and community participation (Sanders and Stappers, 2008; Donetto et al., 2015). The key expected outcome of the workshop was the development of a working algorithm from screening to diagnosis/treatment of breast cancer through active involvement and participation of all stakeholders. 

*The expert group workshop (ICANTREAT)*

The workshop drew on a multidisciplinary, heterogenous stakeholder group that included representatives from relevant fields including clinicians managing breast cancer cases at secondary and tertiary health care facilities; primary care physicians involved in screening and referring the cases; professionals from the field of public health, occupational therapy and communications; program officer involved in prevention and control of non-communicable diseases at the district level (covering a population of 1,200,000); community health workforce; community leaders and breast cancer survivors. The workshop was facilitated by experienced clinicians and researchers from the disciplines of community medicine, public health and health psychology with special interest in oncology. Participants were divided into three task groups with equal representation from above mentioned disciplines to ensure different perspectives and unique contributions were recognised and represented in the discussion of the topic. 

The structured workshop was designed to cover structure, process and outcomes at the level of the individual and the health system. Each group was given the same three topics to discuss and present their conclusions. The discussion topics were: 1) understanding the current process of breast cancer screening, diagnosis and treatment in the local context; 2) developing strategies to improve the existing system; and 3) identification of possible ways to implement strategies to promote use of breast cancer screening and diagnostic facilities. The key areas that were discussed under each of these topics were barriers, facilitators and potential solutions. Once the groups had spent time in discussions, all participants came together in a plenary session to summarize their key conclusions. The groups deliberated the topics in terms of their practical applications, long-term benefits to the patient and improvements of existing screening services.

The workshop concluded with a prioritization exercise to achieve consensus from the whole group. For instance, to understand the current process, the prompts used were, ‘What are the existing facilities for breast cancer screening, diagnosis and treatment? What are the barriers in accessing these facilities?’.

*Analysis*

Responses to the topics were grouped as barriers to uptake of breast cancer screening, perceived facilitators to these barriers and potential solutions to execute these facilitators that would be beneficial to breast cancer patients. The framework by Shiffman and Smith was adapted and modified accordingly to underline the objectives and outcomes of the workshop. This framework was primarily designed to highlight important global health initiatives in order to gather political priority and response (Shiffman and Smith, 2007).

Among the four categories of original framework, we retitled ‘political contexts’ as ‘community health/ public health contexts’. We retained the remaining categories as in the original framework. However, some of the factors under these categories were modified based on the subthemes that emerged from the data. Such inductive subthemes are described as follows and are also notified in Table 1. 

a. Under the category of ‘actor power’, we modified ‘policy community cohesion’, ‘guiding institutions’ and ‘civil society mobilization’ to ‘stakeholder community cohesion’, ‘organizations involved’ and ‘mobilization at grass root level’, respectively. 

b. Under the category of ‘ideas’, we modified ‘internal frame’ to ‘individual level barriers and facilitators’ and ‘external frame’ to ‘system level barriers and facilitators’.

c. Under ‘community health/ public health contexts’, we modified its categories to specifically highlight lack of guidelines for breast cancer screening and referral. We renamed the factors as ‘affordability of screening services’ and ‘dearth of policy guidelines in cancer care pathway’.

## Results

The key discussion areas in the ICANTREAT workshop dealt with individual and system level concerns and the solutions therein. 

*Individual level concerns*

*Prevailing barriers*

Stigma and superstitions governed by cultural inhibitions were described as a stumbling block to the uptake of the screening program. Women are shy to talk about the problem because the disease is perceived to be hereditary and this could have a deleterious effect on the marriage prospects of young girls in the family. Culturally, women in this region prioritize health of other family members over their own. Besides, the prevailing patriarchy accords a precedence to men’s health further preventing them from seeking timely health care. Another argument that found favour with the majority of the participants was the fear of a positive finding which acted as a deterrent to the uptake of screening services. 

*Perceived facilitators*

Collectively it was agreed that the clichéd dictum of creating awareness about the disease and its outcome was still very relevant in overcoming individual level barriers. Enhancing participation of male family members in women’s health was perceived as a possible facilitator and was extensively discussed. Additionally, there were suggestions to involve cancer survivors as community champions and ‘change’ leaders to encourage women’s active participation in preventive health activities. It was anticipated that this would bring in an atmosphere of empowerment and in turn encourage women to overcome denial and actively participate in screening activities. 

*Proposed potential solutions*

Solutions predominantly focused on a comprehensive inclusion of various stakeholders in breast cancer screening and awareness activities. A working, accessible and a dedicated helpline for breast cancer was considered a plausible solution for information dissemination among beneficiaries. It was recognized that changing behaviour would need the involvement of key role models such as celebrities, community champions and ‘easy-to-connect’ community leaders. Keeping in mind the cultural mindset of the local population, active involvement from religious leaders was also deemed appropriate. The statement “It is all about who says it and how it is said” aptly summarizes this strategy.

*System level concerns*

*Prevailing barriers*

Lack of affordable and organized screening facilities especially in the rural areas was considered an important system-level barrier. Non- existent standard protocols for screening and referrals was stated as a further challenge to the already compromised health system with respect to cancer care. The group also agreed that there is currently an absence of coordination between stakeholders in the cancer care pathway, which needs to be addressed. 

*Perceived facilitators*

Training grass root level health workers in community-based screening and cancer health promotion was considered a feasible facilitating factor. This should include a dedicated patient advocate to facilitate the progression from screening to timely referral and follow-up, a crucial element in the cancer care pathway. Increasing engagement with key stakeholders in the cancer care pathway was also believed to be an important enabler. It was proposed to achieve this by putting a community of expertise in place, supported by a virtual platform for engagement. 

*Proposed potential solutions*

In addition to involving grass root level health care workers to enhance community awareness, a patient advocate was highlighted as an important enabler. Influencing government policies by recommending a feasible screening and referral system was emphasized as a key solution. A need to organize such stakeholder meetings at regular intervals to revise and revive individual roles in the cancer care pathway was also stressed. Among other potential solutions, priming social connectors in the community such as beauticians and self-help group leaders, to spread the word about breast cancer and screening, emerged as creative answers to the problem. Given that cancer care is expensive and 80% of health care in India continues to be out of pocket, garnering philanthropic support was considered a necessary step in enhancing affordability and accessibility to breast cancer care.

**Table 1:** Modified Schiffman & Smith’s Framework Highlighting the Objectives and Outcomes of the Workshop

Category	Description	Factors shaping political priority
Issue characteristics	Features of the problem	-Credible indicators a. Breast cancer is the most common cancer among females in India b. Poor patient outcomes as a result of lack of disease awareness and late presentation. - Severity a. India accounts for a high mortality to incidence ratio in comparison to the west. -Effective interventions a. Mapping barriers to uptake of screening services in the region b.Creating a ‘community of expertise’ or multi-stakeholder network that can overcome set- backs in the cancer- care pathway.
Actor power	The strength of the individuals and organizations concerned with the issue	-Stakeholder community cohesion* a. A comprehensive and heterogenous network of stakeholders should be put in place to aid the process from screening to timely referral in order to improve patient outcomes. This is currently lacking in the system. -Leadership a. Involvement of ‘easy to connect’ community leaders, community champions and religious leaders in awareness programmes is important to establish a connection with the local community. -Organisations involved* a. Philanthropic initiatives and participation of private entities as part of ‘corporate social responsibility’ to enable utilization of screening service among the rural population. -Mobilization at grass root level* a. Training of grass root level health workers in screening and referral activities. b. With a multi-stakeholder group in place, any set- backs faced by these workers can be communicated effectively to policy makers and programme officers.
Ideas	The ways in which actors understand and portray the issue	- Individual level barriers, facilitators and potential solutions* a. Important individual level barriers were social stigma and superstitious beliefs associated with breast cancer, lack of participation of men in health issues of women and fear of a positive finding b. Notable facilitators and potential solutions suggested were creating awareness about the disease and engagement of cancer survivors to empower fellow women. - System level barriers and facilitators* a. Important barriers were the dearth of standard guidelines for screening and lack of communication among stakeholders who are involved in the cancer- care pathway. b. Increasing engagement among various stakeholders and involvement of a ‘patient advocate’ were suggested as important facilitators and solutions
Community health/ public health contexts†	The environments in which actors operate	- Affordability of screening services* a. Prevailing lack of affordable screening facilities especially among the rural population needs to be addressed as a priority. - Dearth of policy guidelines in cancer-care pathway* a. There is need to constitute standard guidelines for screening and referral for breast cancer

* † factors and categories modified based on inductive subthemes that emerged from discussions

## Discussion

This workshop was an important step in bringing together key stakeholders to understand various barriers for the uptake of screening services for breast cancer and co-design strategies to address these in rural south India. The results of the workshop provided crucial indicators to the development of practicable, achievable and sustainable interventions including a clinical care pathway. The outcomes of this workshop further resonate with findings from other similar studies especially with respect to active involvement and a community of expertise of stakeholders at various levels (Duggan et al., 2017) as well as the importance of community engagement for better outcomes in the pathway (Musonda, 2018). It became apparent during this workshop that an active and a timely involvement by stakeholders is crucial in ensuring a supportive journey to the beneficiaries. 

The limitations of the current work may include representation from all the key stakeholder groups and the geographical area we worked within and may not be generalizable but these are offset, to some extent, by achieving balance across the stakeholder groups. The workshop was facilitated by a strong team of experienced and early career researchers, who worked to ensure that everyone had a chance to be heard without any one voice dominating the conversation. This co-design workshop also marked the first step in the engagement and development of the ICANTREAT community of expertise, with all participants actively signing up to be part of the initiative. 

This ICANTREAT community of expertise and their support is crucial in the sustainability of the initiative and we propose to set up a virtual community using online platform and create a forum for strengthening the system for cancer care. The evaluation of medium to long-term success needs to be carefully incorporated into the future interventions that build on the findings of this study.
